# Wearability and preference of mouthguard during sport in patients undergoing orthodontic treatment with fixed appliances: a randomized clinical trial

**DOI:** 10.1093/ejo/cjab062

**Published:** 2021-11-08

**Authors:** Aneesh Kalra, Claire Harrington, Gursharan Minhas, Spyridon N Papageorgiou, Martyn T Cobourne

**Affiliations:** 1 Department of Orthodontics, Royal Surrey County Hospital NHS Foundation Trust, Guildford, UK; 2 Centre for Craniofacial Development & Regeneration, Department of Orthodontics, Faculty of Dentistry, Oral & Craniofacial Sciences, King’s College London, UK; 3 Clinic of Orthodontics and Pediatric Dentistry, Center of Dental Medicine, University of Zurich, Switzerland

## Abstract

**Background:**

Orthodontic patients wearing fixed appliances are susceptible to traumatic dental injuries during a wide range of sporting activities. This randomized clinical trial investigated wearability and preference of mouthguards during sporting activities in patients undergoing orthodontic treatment with fixed appliances.

**Methods:**

A prospective three-arm crossover randomized clinical trial conducted in the UK. Thirty patients in active orthodontic treatment with fixed appliances undertaking at least 120 minutes of contact sport per 6–8-week observation period were randomly assigned to one of six mouthguard allocation sequences consisting of three mouthguard types: (MG1) custom-made laboratory constructed, (MG2) mouth-formed OPRO® Gold Braces, and (MG3) pre-fabricated Shock Doctor® Single Brace. Patients completed a nine-outcome 100-mm visual analogue scale (VAS) questionnaire relating to mouthguard wearability during sport. Once feedback was completed, subjects were allocated the next mouthguard in the sequence. At study-end, subjects were asked to identify their preferred mouthguard.

**Results:**

Twenty-four patients (median age = 13; inter-quartile range 12–14.5 years) completed *n* = 72 follow-up questionnaires with most playing rugby union or field hockey. Considering VAS score as a continuous variable, for comfort, stability, hardness, ability to breathe, ability to not cause nausea, and inclination to chew, MG2 performed better than MG3. For categorization of VAS score into low (less than 80 mm) or high (at least 80 mm) wearability, for comfort, stability, ability to not cause nausea, and inclination to chew, MG1 and MG2 also rated superior to MG3. Patients preferred MG1 overall.

**Conclusions:**

This randomized clinical trial found that during contact sport patients in fixed appliances reported superior wearability for custom-made and mouth-formed mouthguards in comparison to pre-fabricated. Overall, patients preferred custom-made mouthguards.

**Clinical trials registration:**

ClinicalTrials.gov: NCT04588831.

## Introduction

Traumatic dental injuries (TDIs) have been described as the fifth-most prevalent disease, with over 1 billion individuals living with the consequences of trauma to their dentition (1). TDIs have an overall prevalence of 18.1 per cent in 12-year-old children with males up to 50 per cent more likely to be affected (2). A number of predisposing factors have been identified in relation to risk of dental trauma, which include increased overjet (3), obesity (4), hooliganism (5), alcohol use (6), and previous dental trauma (7).

Sporting activity is a popular recreational pastime for many children and adults. A number of sports that involve various forms of physical contact carry a risk of trauma to the teeth or associated structures, including football, rugby, basketball, martial arts, boxing, netball, skateboarding, and ‘bat and ball’ sports, such as field hockey, ice hockey, and lacrosse (8). Physical injury can be a frequent consequence of participation in these sports and in the orofacial region, trauma to the maxillary incisor dentition is the most common type (9–12). Traditionally, boys are more likely to be affected than girls but this demographic is changing as participation in all sports by girls and women increases (13, 14).

A mouthguard is an item of protective equipment available for individuals engaging in sporting activity and it is recommended that participants in all sports involving a risk of trauma to the teeth and associated structures should wear a dentally fitted laminated mouthguard during both training and actual games or competition (8). There is some evidence of poor mouthguard acceptance amongst sports players, with a lack of awareness regarding the risks of dental injury during sport and the role of mouthguards in their prevention often cited as a reason amongst coaches, parents, and players themselves (15–17). Further barriers include feelings amongst sports players that mouthguards are unnecessary and uncomfortable (18). Indeed, even in players who are aware that mouthguards can help prevent orofacial injuries, their uptake can be surprisingly low (19, 20) although this appears to change in players who have sustained orofacial injuries during sport (17).

Orthodontic treatment with fixed appliances is commonly carried out in adolescence (21), a period of time with a high incidence of dental injuries in relation to leisure and sporting activities (22, 23). A fixed appliance can provide a further deterrent to wearing a mouthguard because it can compromise the fit, not only through the presence of the appliance itself but also the progressive tooth movement that occurs with treatment (24). Moreover, a fixed appliance can potentially increase the extent of damage following an orofacial injury through debonding of brackets, archwire deformation, and soft tissue laceration (25). There is a lack of evidence-based guidance relating to mouthguard use and fixed appliances; as a result, orthodontists can differ significantly in the advice that they give to patients and parents (26, 27).

There are essentially three types of orthodontic mouthguard available for patients undergoing treatment with fixed appliances: custom-made mouthguards (constructed by a dental technician on a stone cast derived from an impression or intra-oral scan of the recipient’s dentition), simple commercially available thermoformable mouth-formed mouthguards (mass produced and requiring the user to mould them to the dentition prior to use after immersion in hot water, so-called ‘boil and bite’), and simple pre-fabricated commercially available mouthguards (mass produced and designed for an ‘instant fit’ into the mouth). The majority of consultant orthodontists in the UK recommend a custom-made mouthguard (27) and there is some evidence they perform better in laboratory testing (28–32). However, there is little prospective data from orthodontic patients in active treatment in relation to these different mouthguard designs.

This randomized clinical trial has investigated wearability and preference of three mouthguard types during sporting activity in patients undergoing orthodontic treatment with fixed appliances. The null hypothesis was that no difference in wearability and preference existed between the mouthguards investigated in this study.

## Materials and methods

### Trial design

Data for this investigation were gathered from a three-arm crossover randomized clinical trial comparing wearability and preference of different mouthguard designs in orthodontic patients with fixed appliances through feedback using a visual analogue scale (VAS) questionnaire. Trial methodology is reported according to the CONSORT statement (33) with modifications for crossover trials (34). Ethical approval for this study was obtained from the United Kingdom National Research Ethics Service (East of England-Cambridge South REC: 16/EE/0304) and written-informed consent obtained from all patients and their parent/carer/guardian. No changes to methodology occurred after trial commencement. This trial is registered at ClinicalTrials.net (NCT04588831) where the full trial protocol is available.

### Participants, setting, and eligibility criteria

Patients undergoing routine orthodontic treatment with full fixed appliances for a range of malocclusions were recruited from the Orthodontic Department, Royal Surrey County Hospital NHS Foundation Trust, UK. Eligibility for inclusion in the study consisted of the following: 1. 10–18 years of age, 2. undergoing orthodontic treatment with pre-adjusted edgewise fixed appliances, 3. playing at least 120 minutes of sport involving physical contact or risk of injury per 6–8-week observation period (a representative interval between routine fixed appliance adjustments), and 4. no sensory processing disorders. Exclusion criteria included 1. patients with less than 9 months of treatment left, 2. any diagnosis of a sensory processing disorder, and 3. any patients where it was felt that they would not be able to complete the VAS.

### Interventions

**Figure 1.**
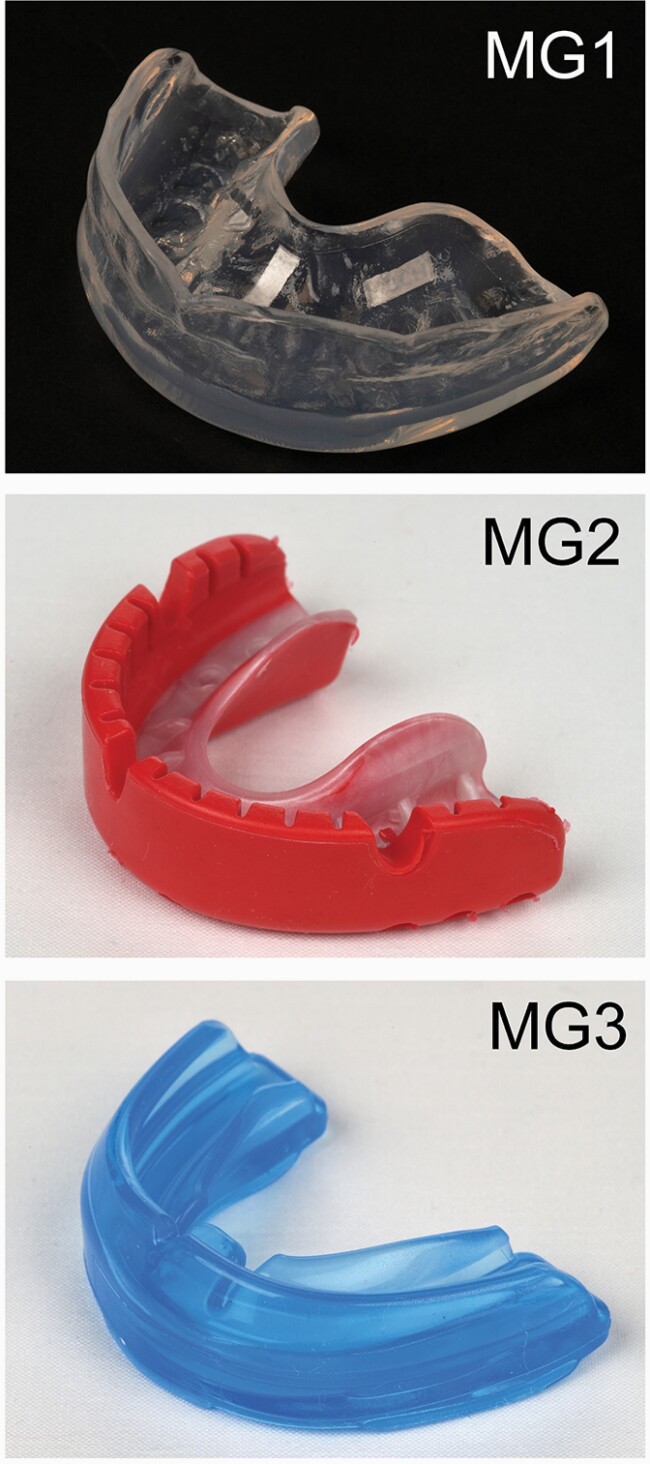
Mouthguard types used in the study. From upper to lower: MG1, custom-made laboratory constructed; MG2, mouth-formed OPRO® Gold Braces; and MG3, pre-fabricated Shock Doctor® Single Brace.

The following three mouthguard types were worn by participants in the study: MG1, custom-made laboratory constructed; MG2, mouth-formed OPRO® Gold Braces (OPRO Ltd, Hemel Hempsted, UK), and MG3, pre-fabricated Shock Doctor® Single Brace (Shock Doctor Inc, Plymouth, Minnesota, USA; Figure 1). The MG1 custom-made mouthguard was constructed from ethylene-vinyl-acetate (EVA) by a laboratory technician using a dental stone model cast (Model Stone White Orthodontic Stone, ISO Type 3, Whipmix, USA) derived from a maxillary alginate impression taken of each patient following recruitment. All MG1 used in this investigation were constructed by a single trained technician (Basingstoke and North Hampshire Hospital, Hampshire Hospitals NHS Foundation Trust) and fabricated to fit around the fixed appliance and dentition. Briefly, a two-layer laminating technique was used incorporating 2 mm followed by 4 mm EVA (Erkoflex, Erkodent, Germany) pressure-formed over the dental cast (35). The mouthguard was constructed with a labial extension to within 2 mm of the vestibular reflection, a rounded labial flange and tapered palatal edge and extension of the palatal flange to within 10 mm of the gingival margin. MG1 was posted direct to patients following construction. The MG2 mouth-formed mouthguard is commercially available and designed for direct moulding around the teeth and alveolar processes by the user according to the manufacturer instructions. It can be re-moulded several times over time and has a durable outer and flexible inner layer made from multiple blends of EVA. It has short fins and a thicker lip bumper to facilitate moulding around fixed appliances. The MG3 pre-fabricated mouthguard is also a commercially available and constructed from 100 per cent medical-grade latex-free silicone, bisphenol-A, and phthalates. It incorporates an ‘ortho-channel’ to aid retention over fixed appliances and no modifications are required by the user before wearing. MG2 and MG3 were purchased direct from the manufacturer.

For each mouthguard in the allocated sequence, patients were given direct verbal instructions on correct fit and wear supplemented by a British Orthodontic Society written patient information leaflet on use of mouthguards (36). The patient was asked to record the amount of time the allocated mouthguard was worn during one specific sport of their choice within the 6–8-week observation period. This was used to determine if a threshold of 120 minutes mouthguard wear had been achieved during sport, allowing the patient to complete a questionnaire relating to mouthguard wearability and then trial the next mouthguard in their randomly allocated sequence. No formal washout period was scheduled between mouthguards.

Patients were asked to complete the nine-item 100 mm VAS validated questionnaire relating to wearability of the allocated mouthguard during sport at each review appointment (*n* = 3 questionnaires in total; Supplementary File 1) (37). The specific outcome variables representing wearability of each mouthguard during sport were Q1 How comfortable was your mouthguard? (0 = uncomfortable; 100 = comfortable); Q2 How bulky did you feel the mouthguard was? (0 = bulky; 100 = not bulky); Q3 How stable in your mouth was the mouthguard? (0 = unstable; 100 = stable); Q4 How hard or soft did you feel the mouthguard was? (0 = soft; 100 = hard); Q5 How difficult was it to breathe with the mouthguard in your mouth? (0 = extreme difficulties breathing; 100 = no difficulties breathing); Q6 How easy or difficult was it to speak with the mouthguard in your mouth? (0 = extreme difficulty; 100 = no difficulty); Q7 How dry did your mouth feel with the mouthguard? (0 = very dry; 100 = no dryness); Q8 Did the mouthguard ever make you feel sick? (0 = felt sick all the time; 100 = never felt sick); and Q9 Did you find that you chewed the mouthguard? (0 = chewed all the time; 100 = never chewed). Completed questionnaires were collected by the principal investigator (AK) direct from each patient and stored with the trial site file in a locked office. Each set of question responses were blinded for mouthguard type and measured directly from the relevant VAS by an independent assessor using a 150 mm plastic ruler as the distance from zero to the point where the subject had made a mark crossing the VAS line. VAS scores were recorded in Microsoft Excel (Microsoft) and transferred into Stata SE 14.0 (StataCorp, College Station, Texas, USA). At study-end, subjects were shown images of the three mouthguards and asked to identify the one that they preferred overall.

### Primary and secondary outcomes

The primary outcome of this trial was mouthguard wearability during participation in sport for patients in fixed appliances based upon nine outcome variables (comfort, bulk, stability, hardness, ability to breathe, speech, mouth dryness, ability to not cause nausea, and ability to induce chewing). These were assessed as the median score for each outcome. The secondary outcome was represented by preference for each mouthguard denoted as the proportion of patients with an answer at least 80 mm on the VAS score for each outcome.

Data from patients who withdrew from the study because they stopped playing their season-based sport were excluded from the analysis. Patients unable to wear any particular mouthguard(s) for the required 120 minutes of sport within the 6–8-week observation period did not complete a VAS questionnaire for that mouthguard and data was collected in an effort to do an intention-to-treat analysis. However, ultimately five patients having received only one-third mouthguards yielded data and these were omitted because no randomized comparison between at least two mouthguards could be done.

### Sample size calculation

A prospective questionnaire-based study investigating orthodontic mouthguards in patients with fixed appliances was not found in the literature. The sample size calculation for this study was therefore based on within-subject variability in responses and performed using G*Power version 3.1.5 (Informer Technologies, Inc.). For an *α*-level of 0.05, a *β*-value of 0.20, and an assumed effect size of 0.4, this study required a total sample size of 22 subjects (providing 66 questionnaires). To allow for dropouts and non-compliance, a total of 30 subjects were recruited.

### Randomization

Participants were randomly assigned to one of six mouthguard allocation sequences composed of the three mouthguard types (MG1, MG2, and MG3). Computer-generated online software (www.random.org) was used to generate the randomization sequence (CH) with participant allocation undertaken independently by AK and GM (allocation-concealment). The study participant identifying number (*n* = 1–30), which held the randomized allocation sequence, was concealed in a sealed, opaque envelope held in a tamper-proof environment under the care of a dedicated orthodontic nurse. Once consented to the study, an envelope was randomly chosen by the participant mediated through the nurse and opened to reveal the sequence of mouthguard allocation.

### Blinding

Blinding was not possible for participants or clinicians. Data were anonymized for statistical analysis.

### Statistical methods (outcomes, measurement reliability, and agreement)

Normality of continuous outcomes was checked through visual plot inspection and formally with the Shapiro–Wilk test. As continuous variables (age, wear time, and VAS scores for each of the nine questions) were not normally distributed, descriptive statistics included medians and inter-quartile ranges (IQRs). The descriptives for categorical variables (gender, sport type, having high VAS score, and mouthguard preference) included absolute and relative frequencies. Generalised Linear regression Modelling (GLM) for the binomial family with log-link using relative risks (RRs) with 95 per cent confidence intervals (CIs) and robust errors to account for clustering within patients was used on the outcome of having a VAS at least 80 mm. Crude differences across mouthguards were checked with Friedman tests (average VAS scores) or GLMs (proportion with VAS at least 80 mm). Initially, crude (univariable) models were constructed with mouthguard category as a sole independent variable. *Post hoc* pairwise comparisons with Holm-Sidak *P*-value corrections for multiple testing were employed after overall statistically significant Friedman tests (with Conover’s test) or after significant GLM models. Afterwards, patient age, gender, and wear time of at least 12 hours, and sport type were added one at a time to check their influence on the coefficient of mouthguard category and retained in the adjusted (multivariable) model if the change-in-estimate was at least 10 per cent (38). The possibility of carry-over effects as well as the impact of the mouthguard assignment order on the primary outcome was assessed in a sensitivity analysis using Friedman tests. All analyses were run in Stata with alpha set at 5 per cent.

To examine measurement reliability and agreement, all VAS scores from the questionnaires were re-measured after 2 weeks. The concordance correlation coefficient (CCC) (39) and Bland–Altman method (40) were used to test intra-examiner reliability and agreement. All data from this trial are deposited at Zenodo (41).

## Results

### Participant flow

**Figure 2.**
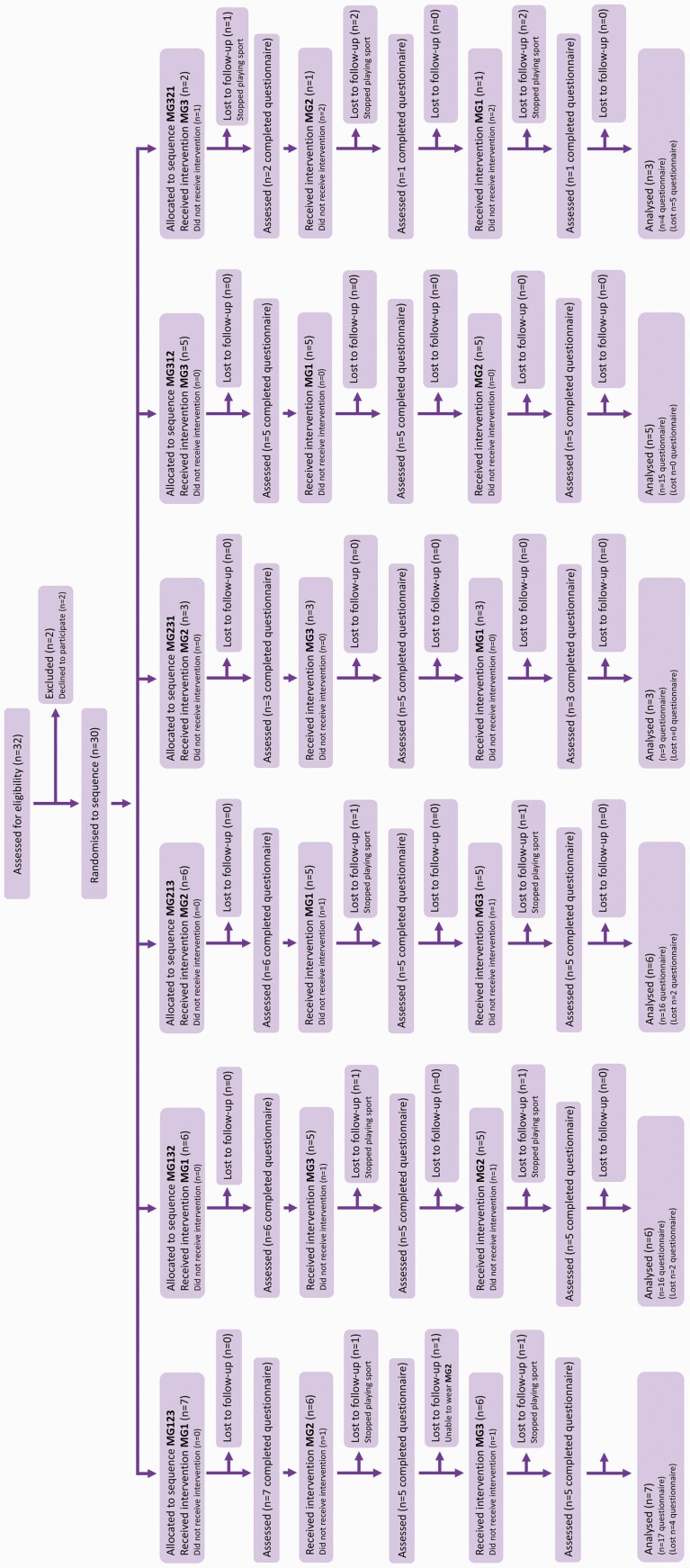
CONSORT flow-diagram of subjects within the trial. (MG1) custom-made laboratory constructed mouthguard, (MG2) mouth-formed OPRO® Gold Braces mouthguard, and (MG3) pre-fabricated Shock Doctor® Single Brace mouthguard.

Thirty patients were recruited between January 2017 and May 2019. A total of 24 patients trialled all three mouthguards comprising 14 males and 10 females with a median age of 13 (IQR 12–14.5) years and completing a total of 72 questionnaires. A CONSORT diagram demonstrating subject flow through the trial is shown in Figure 2 (34). There were (*n* = 5) dropouts related to cessation of sporting activities and (*n* = 1) patient who was unable to wear MG2 and MG3; representing a total loss of (*n* = 13) questionnaires. Median wear time was 12 hours (IQR = 6.8–18.5) with 52 per cent of patients reporting total wear times between 2 and 12 hours and the remaining 48 per cent reporting wear times of greater than 12 hours.

The MG1 custom-made laboratory constructed mouthguard was the preferred choice overall for more than half of trial participants (*n* = 13; 54 per cent), whilst nearly a third preferred the MG2 mouth-formed OPRO® Gold Braces (*n* = 7; 29 per cent) with only *n* = 4 (17 per cent) preferring the MG3 pre-fabricated Shock Doctor® Single Brace mouthguard.

### Sport participation

**Figure 3.**
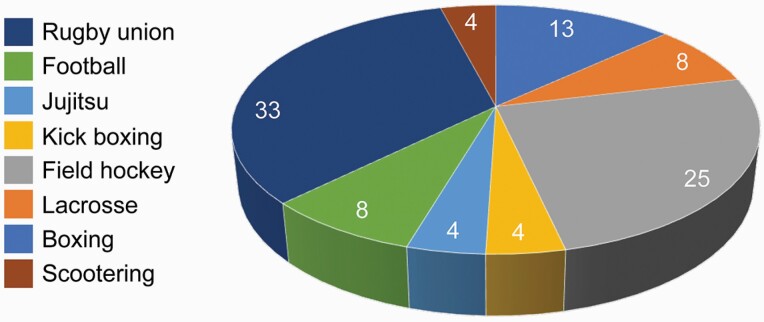
Sport participation amongst the sample (%).

Participants were asked to identify the sport played wearing the mouthguard. Eight different sports were recorded with most patients playing either rugby union (33 per cent) or field hockey (25 per cent; Figure 3).

### Primary and secondary outcomes

**Figure 4.**
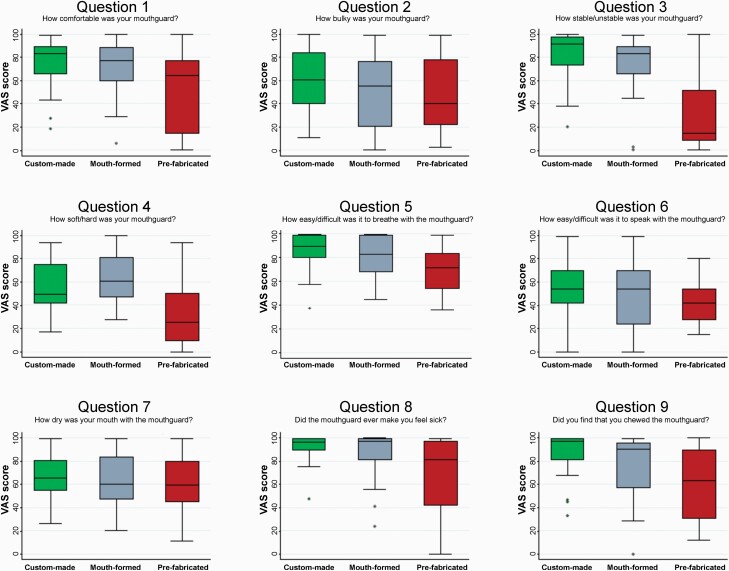
Median visual analogue scale (VAS) scores for the nine questions relating to mouthguard tolerance.

Median VAS scores for each mouthguard associated with each of the nine wearability outcome questions are shown in Table 1 and Figure 4.

**Table 1.** Crude differences amongst mouthguards for median visual analogue scale (VAS) scores and percentage of answers at least 80 mm.

	MG1 custom-made	MG2 mouth-formed OPRO® Gold	MG3 pre-fabricated Shock Doctor®		MG1 custom-made	MG2 mouth-formed OPRO® Gold	MG3 pre-fabricated Shock Doctor®	
	VAS score as a continuous variable			P*	VAS score categorization as high (≥80 mm)			P**
	Median (IQR)				Number of responses with VAS ≥ 80 mm (%)			
Q1 Comfort	83.5 (65.6–89.3)	76.9 (59.6–88.3)	64.4 (14.9–77.4)	0.04	14 (58)	10 (42)	4 (17)	0.03
Q2 Bulk	60.1 (40.1–84.1)	55.0 (20.6–76.5)	40.0 (22.3–77.6)	0.07	7 (29)	5 (21)	6 (25)	0.82
Q3 Stability	91.5 (73.5–97.4)	82.9 (66.0–88.8)	14.3 (8.3–51.8)	0.001	17 (71)	15 (63)	4 (17)	0.02
Q4 Hardness	49.6 (41.5–74.8)	60.9 (47.1–81.1)	25.0 (9.8–50.0)	0.001	5 (21)	6 (25)	1 (4)	0.26
Q5 Ability to breathe	89.0 (79.8–99.0)	82.3 (67.9–98.9)	71.4 (53.9–83.4)	0.03	18 (75)	13 (54)	10 (42)	0.03
Q6 Speech	53.6 (41.8–70.0)	54.1 (23.4–69.9)	41.4 (27.6–53.5)	0.20	3 (13)	5 (21)	1 (4)	0.13
Q7 Mouth dryness	66.0 (54.8–80.5)	60.4 (47.5–84.0)	59.5 (45.5–80.1)	0.21	6 (25)	6 (25)	6 (25)	1.00
Q8 Ability to not cause nausea	96.5 (89.8–99.4)	97.0 (81.5–99.4)	81.0 (42.0–97.3)	0.001	21 (88)	18 (75)	12 (50)	0.02
Q9 Ability to induce chewing	96.9 (81.5–99.1)	90.3 (57.6–95.4)	63.1 (31.0–89.8)	0.04	18 (75)	14 (58)	9 (38)	0.05

IQR, inter-quartile range.

*From Friedman test.

^**^From generalized linear model.

Significant differences in median VAS scores amongst the three mouthguards were found for many of the wearability outcomes (Table 1). Taking VAS score as a continuous variable: for comfort, stability, hardness, ability to breathe, ability to not cause nausea, and inclination to induce chewing, MG1 and MG2 were more wearable than MG3 (Table 1). Pairwise comparisons confirmed that wearability of MG1 and MG2 was better than MG3. MG1 were also more wearable than MG2 in terms of stability, ability to breathe, and inclination to induce chewing, whilst for hardness they were less wearable than MG2 (Supplementary File 2).

For VAS score categorization into low (less than 80 mm) or high (at least 80 mm) wearability: for comfort, stability, ability to breathe, and ability to not cause nausea, differences were also seen amongst the mouthguards (Table 1). Pairwise comparisons indicated that MG1 were more wearable than MG3 for comfort, stability, and ability to not cause nausea. Additionally, MG2 were also more wearable than MG3 for stability (Supplementary File 2).

Table 2 shows differences amongst mouthguards in terms of wearability outcome variables that had high satisfaction (VAS score at least 80 mm) for each outcome (univariable analysis). Patients reporting MG1 as more comfortable and more stable than MG3 were 2.5-fold and 3.3-fold greater, respectively. In addition, patients reported that MG1 were 80 per cent better in terms of their ability to allow easy breathing, 80 per cent less likely to cause nausea and twice as likely to be less inclined to induce chewing compared to the MG3. Patients reporting MG2 as more stable was 2.8-fold greater than MG3. For most questions, MG1 fared better than MG2 (having greater RRs versus MG3), except for hardness and speaking difficulties.

**Table 2.** Generalized linear model univariable analysis on percentage of answers with visual analogue scale (VAS) scores at least 80 mm.

	Q1 Comfort		Q2 Bulk		Q3 Stability		Q4 Hardness		Q5 Ability to breath	
	RR (95% CI)	P	RR (95% CI)	P	RR (95% CI)	P	RR (95% CI)	P	RR (95% CI)	P
**MG1 custom-made**	3.5 (1.3–9.5)	0.01	1.2 (0.5–2.9)	0.74	4.3 (1.6–11.6)	0.005	5.0 (0.6–44.8)	0.15	1.8 (1.1–3.1)	0.03
**MG2 mouth-formed OPRO® Gold**	2.5 (0.8–7.5)	0.10	0.8 (0.3–2.5)	0.75	3.8 (1.4–10.2)	0.01	6.0 (0.7–52.2)	0.10	1.3 (0.7–2.6)	0.45
**MG3 pre-fabricated Shock Doctor®**	Ref		Ref		Ref		Ref		Ref	
	Q6 Speech		Q7 Mouth dryness		Q8 Ability to not cause nausea		Q9 Ability to induce chewing			
**MG1 custom-made**	**RR (95% CI)**	***P***	**RR (95% CI)**	***P***	**RR (95% CI)**	***P***	**RR (95% CI)**	***P***		
**MG2 mouth-formed OPRO® Gold**	3.0 (0.3–30.3)	0.35	1.0 (0.5–1.9)	1.00	1.8 (1.2–2.7)	0.009	2.0 (1.1–3.5)	0.02		
**MG3 pre-fabricated Shock Doctor®**	5.0 (0.8–30.0)	0.08	1.0 (0.5–1.9)	1.00	1.5 (0.9–2.4)	0.09	1.6 (0.9–2.7)	0.11		
MG3	Ref		Ref		Ref		Ref			

CI, confidence interval; Q, question; Ref, reference; RR, relative risk.

Estimation of the limits of agreement between the two outcome assessors demonstrated excellent reliability and reproducibility of the measurement method (Supplementary File 3). CCC showed perfect intra- and almost perfect inter-rater reliability with coefficients of 1.00 and 0.99, respectively.

### Confounding effects

Finally, the potential confounding effects of age, gender, wear time, and participating sport were assessed (Supplementary File 4). Eight different sports were recorded with most patients playing either rugby union or field hockey (see Figure 3) with insufficient numbers playing the remaining sports to detect any differences between mouthguards when adjusting for this potential confounder, therefore only two sports (rugby union versus field hockey) were tested. No evidence of confounding in the trial results was found, since the results of the adjusted (multivariable) analyses were very similar to the crude (univariable) analyses. The only exception being MG1 having a greater advantage in the adjusted analysis over MG3 for comfort and stability compared to the crude analysis, after controlling for sporting activity differences (Supplementary File 5). Sensitivity analysis according to the order with which the mouthguards were assigned to the patients indicated no significant differences (Supplementary File 6).

### Harms

There were no harms reported for any component of this trial.

## Discussion

This randomized crossover clinical trial has investigated wearability and preference of three common mouthguard designs in a cohort of patients undergoing fixed appliance orthodontic treatment and participating in contact sports. This is the first prospective investigation of mouthguard design in orthodontic patients. It is important for healthcare professionals to provide evidence-based recommendations to patients and this investigation provides high-level evidence.

Most patients preferred the MG1 custom-made mouthguard when compared to MG2 mouth-formed and MG3 pre-fabricated, which is consistent with previous retrospective analyses (42, 43). This outcome is perhaps to be expected, given that custom-made mouthguards are made bespoke for each patient. Perceived comfort of the mouthguard by the user is a critical component influencing compliance (37) and mouthguards that are individually crafted by trained technicians and fitted by general dentists or orthodontists who can address issues such as stability, bulkiness, and retention through prescription and adjustment without sacrificing protective features are likely to perform better. Although no studies have investigated the effects of prolonged mouthguard wear on orthodontic tooth movement, some orthodontists have expressed concerns that custom-made mouthguards may be too adaptive and consequently prevent desired tooth movement during treatment (26). The patients in this study were at different stages of fixed appliance orthodontic treatment and were only asked to trial each mouthguard between two adjustment appointments. Any potential mouthguard effect on tooth movement was therefore unlikely but was not formally not assessed.

Despite the evidence of orthodontic custom-made mouthguards demonstrating superior wearability, commercially available mouthguards are more conveniently obtained and can be fitted without the intervention of a dental professional. However, more than half of UK orthodontic consultants have the facilities to fabricate custom-made mouthguards but many do not routinely recommend them because of the time and resources required to produce them, the knowledge that pre-fabricated and mouth-formed mouthguards can be readily purchased outside the hospital and a perceived lack of effectiveness (27). Other factors that influence choice of mouthguard include the type of sport played. OPRO® supply custom-made mouthguards in the UK to athletes in partner teams including England Rugby, England Rugby League, British Basketball, and England Boxing. These recommendations transmit to junior levels and influence orthodontic athletes mouthguard choices, despite poor evidence for the most acceptable mouthguard type for these individuals.

A VAS questionnaire was used to measure participant feedback, which is a frequently used tool to measure satisfaction levels in healthcare research. Here, we used a questionnaire developed as part of an investigation into the problems associated with the wearing of mouthguards in rugby players (37). It included questions relating to mouthguard wearability and represents a reliable measure of subject preference in mouthguard choice (44–46).

In a randomized crossover design, participants normally alternate between allocated interventions after a washout period. Having the same set of participants is advantageous because they act as their own controls, which means a requirement for smaller numbers of participants in comparison to parallel-group studies. The risk of not employing a washout period may increase carry-over effects between evaluation phases, introducing bias (47). The carry-over effect in this study was memory, which is difficult to control but in this type of experimental design it would be unethical to stipulate a formal washout period between mouthguards, because this would require subjects to either desist from contact sport for a period of time or engage in it without wearing a mouthguard. In this trial, there were a number of dropouts related to seasonal cessation of sporting activities. Dropouts leading to incomplete data collection can have a larger impact on crossover trials compared to parallel-group designs. Dealing with dropouts is difficult and subjects who only complete part of the study contribute little to the analysis, because missing data from one intervention precludes within-subject comparison (48). However, these subjects remained in the trial on an intention-to-treat basis and over-recruitment meant that the sample remained above that required by the formal sample size calculation.

This randomized clinical trial found that in orthodontic patients with fixed appliances participating in contact sports, custom-made and mouth-formed mouthguards provided a higher level of wearability than pre-fabricated types. Overall, patients preferred the custom-made type. The use of a custom-made or mouth-formed mouthguard is recommended for any individual playing contact sport during treatment with fixed appliances.

## Supplementary Material

cjab062_suppl_Supplementary_File_1

cjab062_suppl_Supplementary_File_2

cjab062_suppl_Supplementary_File_3

cjab062_suppl_Supplementary_File_4

cjab062_suppl_Supplementary_File_5

cjab062_suppl_Supplementary_File_6

## Data Availability

The data underlying this article are available at Zenodo: http://doi.org/10.5281/zenodo.40584792020 (31).
