# Supplementary material for: Wearability and preference of mouthguard during sport in patients undergoing orthodontic treatment with fixed appliances: a randomized clinical trial
Source: Eur J Orthod. 2021 Nov 8;44(1):101–9. doi: 10.1093/ejo/cjab062 (PMC8789322; doi:10.1093/ejo/cjab062)
Supplement: cjab062_suppl_Supplementary_File_1 [file cjab062_suppl_supplementary_file_1.pdf]

# Comfort and Wearability of Orthodontic Mouthguards in comparison to Custom-made Mouthguards: A Randomised Clinical Trial

CASE REPORT BOOKLET V3.0 DATED 3/11/2016

**PRIVATE & CONFIDENTIAL**

STUDY patient identifier:

……………………….

# ELIGIBILITY CRITERIA

Patient is only eligible If **Yes is ticked for all inclusion criteria and No is ticked for all exclusion criteria**

**Study** ID: …………………….. Patients Initials: ………

## Eligibility - Inclusion Criteria

*Source Data Refer to Eligibility Criteria Form filed in patient notes/case notes*

Undergoing fixed appliance treatment for a minimum period of 9 months Y ☐

Plays a sport where use of a mouthguard is recommended Y ☐

Plays at least 120 minutes of sport in 6-8 week period Y ☐

Able to complete a VAS questionnaire Y ☐

Not diagnosed with a sensory processing disorder (SPD) Y ☐

## Eligibility - Exclusion Criteria

*Source Data Refer to Eligibility Criteria Form filed in patient notes/case notes*

**Does not play a sport that recommends the use of a Mouthguard** N ☐

**Undergoing orthodontic treatment which is not fixed appliances** N ☐

**Has Less than 9 Months of fixed appliance treatment left** N ☐

**Has Been diagnosed with a sensory processing disorder** N ☐

**Patient unable to complete a VAS questionnaire** N ☐

## Eligibility - Confirmation

*Source Data Refer to Eligibility Criteria Form filed in patient notes/case notes*

Date Eligibility Confirmed ☐☐/☐☐/☐☐

Confirmed By ______________________

CRF completed By (name): ______________________

Signature: ______________________

Date Completed ☐☐/☐☐/☐☐

Comfort and Wearability of Orthodontic Mouthguards: an RCT

**PRIVATE & CONFIDENTIAL**

**Study** patient identifier: …………………….. Patients Initials: ………



**Randomisation**

*Source Data Refer to case notes*

**Date of Randomisation** ☐☐/☐☐/☐☐

**Group 1: Custom-made Mouthguard** ☐

**Group 2: Orthodontic Mouthguard; Boil and Bite** ☐

**Group 3: Orthodontic Mouthguard; Instant fit** ☐

**Randomisation performed by:** ________________________________

CRF completed By (name): ______________________

Signature: ______________________

Date Completed ☐☐/☐☐/☐☐

# Baseline data

*Source Data Refer to case report form*

**Gender**

Male ☐ Female ☐

Age ☐☐

Concomitant Medication(s);

____________________________

____________________________

____________________________

____________________________

CRF completed ______________________

Signature: ______________________

Date Completed ☐☐/☐☐/☐☐

# First planned Mouthguard Allocation

The Patient will be allocated their first Mouthguard and asked to wear for a period of 6-8 weeks.

Date of visit ☐☐/☐☐/☐☐

They will then be allocated their **first** Mouthguard and a Diary to complete

CRF completed ____________________

Signature: ____________________

Date Completed ☐☐/☐☐/☐☐

# Second planned visit

*Source Data Refer the VAS questionnaire .* Data will be based on the subject completing the questionnaire at their 6-8 review appointment which will include a series of questions and response via a VAS of 100mm line. The patient will be asked to mark a point along the line, which corresponds to how they feel best answers the question regarding the comfort and wearability of the mouthguard of their 1st mouthguard

Date of visit ☐☐/☐☐/☐☐

Time VAS questionnaire completed ☐☐.☐☐ hrs

They will then be allocated their **second** Mouthguard and a new Diary to complete

CRF completed ____________________

Signature: ____________________

Date Completed ☐☐/☐☐/☐☐

Study ID: …………………….. Patients Initials: ………

# Questionnaire

Study patient identifier:
Mouthguard type:

1) How comfortable was your mouthguard?

______________________________________________

Uncomfortable                                        Comfortable

2) How Bulky did you feel the mouthguard was?

______________________________________________

Bulky                                        Not Bulky

3) How stable in your mouth was the mouthguard

______________________________________________

Unstable                                        Stable

4) How hard or soft did you feel the mouthguard was?

______________________________________________

Soft                                        Hard

5) How difficult was it to breathe with the mouthguard whilst doing sports

_______________________________________________

Extreme difficulties breathing | No difficulties breathing

6) How easy or difficult was it to speak with the mouthguard in?

_______________________________________________

Extreme Difficulties Speaking | No difficulties Speaking

7) How dry did you mouth feel whilst wearing the mouthguard

_______________________________________________

Very Dry | No Dryness

8) Did the mouthguard ever make you feel sick

_______________________________________________

Felt sick all the time | Never felt sick

9) Did you find that you chewed the mouthguard

_______________________________________________

Chewed all the time | Never chewed

# Third planned visit

*Source Data Refer the VAS questionnaire . Data will be based on* the subject completing the questionnaire at their 6-8 review appointment which will include a series of questions and response via a VAS of 100mm line. The patient will be asked to mark a point along the line, which corresponds to how they feel best answers the question regarding the comfort and wearability of the mouthguard of their 2nd mouthguard

Date of visit ☐☐/☐☐/☐☐

Time VAS questionnaire completed ☐☐.☐☐ hrs

The participant will then be allocated their **third** mouthguard and a new diary

CRF completed ____________________

Signature: ____________________

Date Completed ☐☐/☐☐/☐☐

Study ID: …………………….. Patients Initials: ………

# Questionnaire

Study patient identifier:
Mouthguard type:

1) How comfortable was your mouthguard?

____________________________________________

Uncomfortable Comfortable

2) How Bulky did you feel the mouthguard was?

____________________________________________

Bulky Not Bulky

3) How stable in your mouth was the mouthguard

____________________________________________

Unstable Stable

4) How hard or soft did you feel the mouthguard was?

____________________________________________

Soft Hard

5) How difficult was it to breathe with the mouthguard whilst doing sports

____________________________________________

Extreme difficulties breathing — No difficulties breathing

6) How easy or difficult was it to speak with the mouthguard in?

____________________________________________

Extreme Difficulties Speaking — No difficulties Speaking

7) How dry did you mouth feel whilst wearing the mouthguard

____________________________________________

Very Dry — No Dryness

8) Did the mouthguard ever make you feel sick

____________________________________________

Felt sick all the time — Never felt sick

9) Did you find that you chewed the mouthguard

____________________________________________

Chewed all the time — Never chewed

# Fourth planned visit

*Source Data Refer the VAS questionnaire . Data will be based on* the subject completing the questionnaire at their 6-8 review appointment which will include a series of questions and response via a VAS of 100mm line. The patient will be asked to mark a point along the line, which corresponds to how they feel best answers the question regarding the comfort and wearability of the mouthguard of their 3rd mouthguard

Date of visit ☐☐/☐☐/☐☐

Time VAS questionnaire completed ☐☐.☐☐ hrs

The patient will be asked a final question regarding their preference of which mouthguard is most comfortable and wearable.

This now the end of the trial

CRF completed ____________________

Signature: ____________________

Date Completed ☐☐/☐☐/☐☐

Study ID: …………………….. Patients Initials: ………

# Questionnaire

Study patient identifier:
Mouthguard type:

1) How comfortable was your mouthguard?

______________________________________________

Uncomfortable Comfortable

2) How Bulky did you feel the mouthguard was?

______________________________________________

Bulky Not Bulky

3) How stable in your mouth was the mouthguard

______________________________________________

Unstable Stable

4) How hard or soft did you feel the mouthguard was?

______________________________________________

Soft Hard

5) How difficult was it to breathe with the mouthguard whilst doing sports

______________________________________________

Extreme difficulties breathing | No difficulties breathing

6) How easy or difficult was it to speak with the mouthguard in?

______________________________________________

Extreme Difficulties Speaking | No difficulties Speaking

7) How dry did you mouth feel whilst wearing the mouthguard

______________________________________________

Very Dry | No Dryness

8) Did the mouthguard ever make you feel sick

______________________________________________

Felt sick all the time | Never felt sick

9) Did you find that you chewed the mouthguard

______________________________________________

Chewed all the time | Never chewed

## **FINAL QUESTION**

10) Which Mouthguard do you prefer (Please tick)?

- Custom-made mouthguard ☐
- Orthodontic Instant Fit Mouthguard ☐
- Orthodontic Boil and Bite Mouthgard ☐
