# Supplementary material for: Wearability and preference of mouthguard during sport in patients undergoing orthodontic treatment with fixed appliances: a randomized clinical trial
Source: Eur J Orthod. 2021 Nov 8;44(1):101–9. doi: 10.1093/ejo/cjab062 (PMC8789322; doi:10.1093/ejo/cjab062)
Supplement: cjab062_suppl_Supplementary_File_2 [file cjab062_suppl_supplementary_file_2.docx]

**Supplementary File 2** Pairwise comparisons for mean VAS score (after a statistically significant Friedman test; here with Conover’s test) and for the percentage of answers ≥80 mm (after a statistically significant GLM model); both with Holm-Sidak correction of p values for multiple testing.

|  |  | **Average VAS score** | | | **% answers ≥80mm** | | | |
| --- | --- | --- | --- | --- | --- | --- | --- | --- |
|  |  | **MG1** | **MG2** | **MG3** | **MG1** | **MG2** | **MG3** |  |
| Q1 | **MG1** | - | - | - | - | - | - |  |
|  | **MG2** | NC  P=1.00 | - | - | NC  P=0.60 | - | - |  |
|  | **MG3** | Δ=+19.5  P<0.001 | Δ=+16.5  P <0.001 | - | Δ=+41.7%  P=0.04 | NC  P=0.28 | - |  |
|  |  |  |  |  |  |  |  |  |
| Q3 | **MG1** |  |  |  |  |  |  |  |
|  | **MG2** | Δ=+7.8  P<0.001 |  |  | NC  P=0.81 |  |  |  |
|  | **MG3** | Δ=+63.5  P<0.001 | Δ=+60.1  P<0.001 |  | Δ=+54.2%  P=0.01 | Δ=+45.8%  P=0.03 |  |  |
|  |  |  |  |  |  |  |  |  |
| Q4 | **MG1** |  |  |  | - | - | - |  |
|  | **MG2** | Δ=-5.4  P<0.001 |  |  | - | - | - |  |
|  | **MG3** | Δ=+18.6  P<0.001 | Δ=+21.5  P<0.001 |  | - | - | - |  |
|  |  |  |  |  |  |  |  |  |
| Q5 | **MG1** |  |  |  |  |  |  |  |
|  | **MG2** | Δ=+4.0  P=0.002 |  |  | NC  P=0.37 |  |  |  |
|  | **MG3** | Δ=+13.9  P<0.001 | Δ=+16.0  P<0.001 |  | NC  P=0.08 | NC  P=0.83 |  |  |
|  |  |  |  |  |  |  |  |  |
| Q8 | **MG1** |  |  |  |  |  |  |  |
|  | **MG2** | NC  P=0.15 |  |  | NC  P=0.61 |  |  |  |
|  | **MG3** | Δ=+4.0  P<0.001 | Δ=+5.4  P<0.001 |  | Δ=+37.5%  P=0.03 | NC  P=0.25 |  |  |
|  |  |  |  |  |  |  |  |  |
| Q9 | **MG1** |  |  |  | - | - | - |  |
|  | **MG2** | Δ=+3.9  P<0.001 |  |  | - | - | - |  |
|  | **MG3** | Δ=+19.5  P<0.001 | Δ=+13.9  P=0.001 |  | - | - | - |  |

*Δ, median difference or difference of proportions (column-row); MG1, Custom-fitted; MG2, Mouth-formed; MG3, Pre-fabricated; NC, not calculated; Q, question*
