# Supplementary material for: Wearability and preference of mouthguard during sport in patients undergoing orthodontic treatment with fixed appliances: a randomized clinical trial
Source: Eur J Orthod. 2021 Nov 8;44(1):101–9. doi: 10.1093/ejo/cjab062 (PMC8789322; doi:10.1093/ejo/cjab062)
Supplement: cjab062_suppl_Supplementary_File_3 [file cjab062_suppl_supplementary_file_3.docx]

**Supplementary File 3** Reliability-agreement of repeated measurements.

|  | **Average Difference (95% LoA)** |  | **CCC (95% CI)** |
| --- | --- | --- | --- |
| Assessor A: T₁ v T₂ | 0.22 (-0.94, 1.37) |  | 1.00 (1.00, 1.00) |
| Assessor B: T₁ v T₂ | 0.07 (-1.08, 1.23) |  | 1.00 (1.00, 1.00) |
| Both assessors: T₁ v T₂ | 0.14 (-1.02, 1.31) |  | 1.00 (1.00, 1.00) |
|  |  |  |  |
| T₁: Assessor A v B | -0.31 (-2.03, 1.41) |  | 0.99 (0.99, 1.00) |
| T₂: Assessor A v B | -0.45 (-2.22, 1.32) |  | 0.99 (0.99, 0.99) |
| T₁ / T₂: Assessor A v B | -0.38 (-2.13, 1.37) |  | 0.99 (0.99, 0.99) |

*CCC, concordance correlation coefficient; CI, confidence interval; LoA, limits of agreement.*
