# Supplementary material for: Wearability and preference of mouthguard during sport in patients undergoing orthodontic treatment with fixed appliances: a randomized clinical trial
Source: Eur J Orthod. 2021 Nov 8;44(1):101–9. doi: 10.1093/ejo/cjab062 (PMC8789322; doi:10.1093/ejo/cjab062)
Supplement: cjab062_suppl_Supplementary_File_4 [file cjab062_suppl_supplementary_file_4.docx]

**Supplementary File 4** Selection of confounders to use in the adjusted analyses using the change-in-estimate criterion. Green highlighted confounders are ultimately retained (* with only rugby versus hockey as catagories due to the small number and widespread distribution of other sports)

| **Q** |  | **Crude** | **With age** | **With male** | **With wear** | **With sport*** |
| --- | --- | --- | --- | --- | --- | --- |
| Q1 | Estimate | -0.5370 | -0.5239 | -0.6191 | -0.5260 | -0.9454 |
|  | CIE | - | 2.4% | -15.3% | 2.0% | 76.1% |
|  |  |  |  |  |  |  |
| Q2 | Estimate | -0.0858 | -0.0797 | -0.0742 | -0.0804 | 0.0335 |
|  | CIE | - | 7.1% | 13.5% | 6.3% | <-100.00% |
|  |  |  |  |  |  |  |
| Q3 | Estimate | -0.4935 | -0.4960 | -0.5101 | -0.4357 | -0.6490 |
|  | CIE | - | -0.5% | -3.4% | 11.7% | 31.5% |
|  |  |  |  |  |  |  |
| Q4 | Estimate | -0.4941 | -0.4896 | -0.4998 | -0.4965 | -0.0681 |
|  | CIE | - | 0.9% | -1.2% | -0.5% | -86.2% |
|  |  |  |  |  |  |  |
| Q5 | Estimate | -0.3007 | -0.3130 | -0.2407 | -0.3020 | -0.3907 |
|  | CIE | - | -4.1% | 20.0% | -0.4% | 29.9% |
|  |  |  |  |  |  |  |
| Q6 | Estimate | -0.3235 | -0.3222 | -0.3529 | -0.1863 | -0.2911 |
|  | CIE | - | 0.4% | -9.1% | 42.4% | -10.0% |
|  |  |  |  |  |  |  |
| Q7 | Estimate | 0.0000 | 0.0888 | 0.0000 | 0.0106 | -0.0467 |
|  | CIE | - | <-100.0% | 94.8% | <-100.0% | <-100.0% |
|  |  |  |  |  |  |  |
| Q8 | Estimate | -0.9927 | -1.0165 | -0.9929 | -0.8754 | -0.3172 |
|  | CIE | - | -2.4% | 0.0% | 11.8% | -68.1% |
|  |  |  |  |  |  |  |
| Q9 | Estimate | -0.3232 | -0.3255 | -0.3040 | -0.3036 | -0.0986 |
|  | CIE | - | -0.7% | 5.9% | 6.1% | -69.5% |

*CIE, change in estimate; Q, question.*

** with only rugby versus hockey as categories due to the small number and wide spread of other sports.*
