# Supplementary material for: Wearability and preference of mouthguard during sport in patients undergoing orthodontic treatment with fixed appliances: a randomized clinical trial
Source: Eur J Orthod. 2021 Nov 8;44(1):101–9. doi: 10.1093/ejo/cjab062 (PMC8789322; doi:10.1093/ejo/cjab062)
Supplement: cjab062_suppl_Supplementary_File_5 [file cjab062_suppl_supplementary_file_5.docx]

**Supplementary File 5** Generalized linear mode (multivariable) analyses adjusting for confounders retained from Supplementary File 4

|  |  | **Q1** | | **Q2** | | **Q3** | | **Q5** | | **Q6** | | **Q7** | | **Q8** | |  |
| --- | --- | --- | --- | --- | --- | --- | --- | --- | --- | --- | --- | --- | --- | --- | --- | --- |
| **Adjusting for** | **Category** | **RR**  **(95% CI)** | **P** | **RR**  **(95% CI)** | **P** | **RR**  **(95% CI)** | **P** | **RR**  **(95% CI)** | **P** | **RR**  **(95% CI)** | **P** | **RR**  **(95% CI)** | **P** | **RR**  **(95% CI)** | **P** | |
| Nothing (crude) | MG1 | 3.5  (1.3-9.5) | 0.01 | 1.2  (0.5-2.9) | 0.74 | 4.3  (1.6-11.6) | 0.005 | 1.8  (1.1-3.1) | 0.03 | 3.0  (0.3-30.3) | 0.35 | 1.0  (0.5-1.9) | 1.00 | 1.8  (1.2-2.7) | 0.009 | |
|  | MG2 | 2.5  (0.8-7.5) | 0.10 | 0.8  (0.3-2.5) | 0.75 | 3.8  (1.4-10.2) | 0.01 | 1.3  (0.7-2.6) | 0.45 | 5.0  (0.8-30.0) | 0.08 | 1.0  (0.5-1.9) | 1.00 | 1.5  (0.9-2.4) | 0.09 | |
|  | MG3 | Ref |  | Ref |  | Ref |  | Ref |  | Ref |  | Ref |  | Ref |  | |
|  |  |  |  |  |  |  |  |  |  |  |  |  |  |  |  | |
| Age | MG1 |  |  |  |  |  |  | 1.6  (1.0-2.6) | 0.06 |  |  | 0.8  (0.4-1.9) | 0.65 |  |  | |
|  | MG2 |  |  |  |  |  |  | 1.2  (0.6-2.4) | 0.51 |  |  | 1.0  (0.6-1.7) | 0.96 |  |  | |
|  | MG3 |  |  |  |  |  |  | Ref |  |  |  | Ref |  |  |  | |
|  |  |  |  |  |  |  |  |  |  |  |  |  |  |  |  | |
| Male | MG1 | 3.9  (1.5-10.5) | 0.006 | 1.1  (0.5-2.9) | 0.78 |  |  |  |  |  |  | 1.0  (0.5-1.9) | 1.00 |  |  | |
|  | MG2 | 2.4  (0.8-7.3) | 0.12 | 0.8  (0.3-2.5) | 0.73 |  |  |  |  |  |  | 1.0  (0.5-2.0) | 0.93 |  |  | |
|  | MG3 | Ref |  | Ref |  |  |  |  |  |  |  | Ref |  |  |  | |
|  |  |  |  |  |  |  |  |  |  |  |  |  |  |  |  | |
| Wear | MG1 |  |  |  |  | 3.9  (1.4-10.5) | 0.008 |  |  | 2.1  (0.2-21.4) | 0.54 | 1.0  (0.5-1.9) | 0.95 | 1.6  (1.1-2.4) | 0.02 | |
|  | MG2 |  |  |  |  | 3.6  (1.3-9.9) | 0.01 |  |  | 3.3  (0.5-20.2) | 0.20 | 1.0  (0.5-1.9) | 0.99 | 1.4  (0.9-2.3) | 0.12 | |
|  | MG3 |  |  |  |  | Ref |  |  |  | Ref |  | Ref |  | Ref |  | |
|  |  |  |  |  |  |  |  |  |  |  |  |  |  |  |  | |
| Sport | MG1 | 5.7  (1.4-23.5) | 0.02 | 0.9  (0.2, 4.0) | 0.93 | 5.7  (1.3-24.1) | 0.02 | 2.3  (1.1-5.1) | 0.04 |  |  | 1.1  (0.5-2.4) | 0.81 | 2.0  (1.1-3.7) | 0.03 | |
|  | MG2 | 2.0  (0.3, 11.8) | 0.45 | 0.8  (0.2, 3.1) | 0.76 | 4.0  (1.0-16.7) | 0.06 | 1.7  (0.6-4.5) | 0.28 |  |  | 1.1  (0.5-2.4) | 0.81 | 1.5  (0.8-2.7) | 0.17 | |
|  | MG3 | Ref |  | Ref |  | Ref |  | Ref |  |  |  | Ref |  | Ref |  | |

*CI, confidence interval; MG1, Custom-fitted mouthguard; MG2, Mouth-formed mouthguard; MG3, Pre-fabricated mouthguard; Q, question; Ref, reference; RR, relative risk.*
