# Supplementary material for: Wearability and preference of mouthguard during sport in patients undergoing orthodontic treatment with fixed appliances: a randomized clinical trial
Source: Eur J Orthod. 2021 Nov 8;44(1):101–9. doi: 10.1093/ejo/cjab062 (PMC8789322; doi:10.1093/ejo/cjab062)
Supplement: cjab062_suppl_Supplementary_File_6 [file cjab062_suppl_supplementary_file_6.docx]

**Supplementary File 6** Sensitivity analysis of median VAS scores for each question according to the order with which the three mouthguards were distributed

| **Answer to variable** | **n** | **Median (IQR)** | **P across mouthguard assignment order (Friedman)** |
| --- | --- | --- | --- |
| Q1 – comfort | 72 | 75.4 (52.1-85.8) | 0.67 |
| Q2 – bulkiness | 72 | 48.5 (30.3-80.0) | 0.27 |
| Q3 – stability | 72 | 79.3 (23.5-90.4) | 0.71 |
| Q4 – hardness | 72 | 48.3 (33.8-66.8) | 0.43 |
| Q5 – breathing difficulties | 72 | 83.0 (67.3-98.4) | 0.57 |
| Q6 – speaking difficulties | 72 | 48.3 (31.6-99.3) | 0.08 |
| Q7 – oral dryness | 72 | 63.5 (47.9-81.0) | 0.15 |
| Q8 – nausea | 72 | 95.9 (77.3-99.1) | 0.53 |
| Q9 – inclination to chew | 72 | 86.8 (49.0-98.1) | 0.61 |

*IQR, interquartile range; Q, question*
